# “Musical co-creation”? Exploring health-promoting potentials on the use of musical and interactive tangibles for families with children with disabilities

**DOI:** 10.3402/qhw.v8i0.20704

**Published:** 2013-08-07

**Authors:** Karette Stensæth

**Affiliations:** Centre for Music and Health/Norwegian Academy of Music, Oslo, Norway

**Keywords:** Co-creation, universal design, participation, tangible interaction, Information and Communications Technology, disability, music, health

## Abstract

The point of departure in this text is the ongoing qualitative interdisciplinary research project RHYME (www.RHYME.no), which addresses the lack of health-promoting interactive and musical Information and Communications Technology (ICT) for families with children with severe disabilities. The project explores a new treatment paradigm based on collaborative, tangible, interactive net-based musical “smart things” with multimedia capabilities. The goal in RHYME is twofold: (1) to reduce isolation and passivity, and (2) to promote health and well-being. *Co-creation* is suggested as a possible path to achieving these goals, by evoking feelings, for example, or accommodating the needs to act and to create social relations; co-creation also motivates users to communicate and collaborate within (new) social relations. This article engages co-creation by incorporating aspects connected to interaction design and the field of music and health. Empirical observations will be referred to. The research question is as follows: What might co-creation imply for families of children with disabilities when musical and interactive tangibles are used as health-promoting implements?

To set the tone, and before introducing the RHYME project and going into the theoretical discussion, this article will start out by presenting glimpses of some live images from the initial participant actions. The following two vignettes describe how two of the six children with disabilities from the participating families in RHYME approach and explore the collaborative, tangible, interactive net-based musical “smart things” with multimedia capabilities (from now on called the co-creative tangibles (CCTs). Both of them are happy and energetic children who show a lot of joy in attending music activities and playing with computers. The children’s cognitive levels are 1–3 years; they both have physical and mental disabilities as well as challenges connected to the autistic spectrum. Two adult women, who know them very well from school, assist the children in their first meetings with the CCTs, which proceeded in a prepared observation room in the children’s school environment.^1^

## Vignettes

*The 11-year-old boy, who is energetic and has few words, enters the observation room for the first time with a woman in her 40s. She assists him, and they hold hands. The boy, whose walk is stiff and slow, gesticulates a lot with his free hand, as if he is helping himself to balance while simultaneously communicating with the close other. They both look at the many orange and black pillow-like things, the CCTs, which are laying around on a carpet on the floor in front of them. They see that the CCTs are shaped as small pyramids and appear in various sizes. The boy and the woman look at each other and smile. He then sits down in the midst of the pillows and soon the close other sits down too, as a response to what she reads as his invitation. The boy starts exploring the pillows while sitting near the woman. She stays still and waits. She watches him and what he does and is there for him and with him. He picks two of the CCTs up: Are they heavy? He then starts to bend and push them … and is slightly surprised when musical sounds suddenly become audible in the pillows. He looks at the woman with an open mouth. He then smiles and taps with his hand on his knee, as if saying: “That was funny!” He listens attentively as he continues to play with the pillows, and when he hears noise music he suddenly becomes aware of the graphics that appear on the screen behind him. He sees many colors, red, yellow, black, etc. He also sees that the colors create patterns on the screen while they move dynamically along with the sound of the music. Then he starts to pay attention to the rhythmic drum playing sounding from the pillows. This music arouses him. He lies down on the floor and starts to “play” drums on his stomach, tapping it like a drum skin. Again, he looks at the woman and smiles. As if accompanying the rhythmical jazz music sounding from the pillows now, the boy intensifies his actions: He gets his body up in a half right position, picks up one of the big pillows, balances on his knees and leans his upper body backwards, simulating that he “falls” along with the fast saxophone improvization which is moving in a downward direction. He then throws the big pillow towards the woman. She is not able to catch it, and instead the pillow lands on the floor with the noise of glass breaking. The boy laughs happily out loud … while looking at the woman, who laughs back to recognize his feelings, and says: “Do you think it is fun to play with these pillows?”*

*A 50-year-old woman lifts the 15-year-old-girl with disabilities up from her wheel chair and places her on the carpet in the middle of the floor. The woman makes sure that two of the big pillows (CCTs) support the girl’s back. The girl, who has severe disabilities and no words but who communicates through a selected repertoire of sounds and finger sign language, sits with her legs crossed. Opposite from her the woman sits down with her legs under her. Around them they see several CCTs, and soon the woman grabs two of the pillows, giving one to the girl and keeping one for herself. They have been at the observation room once before, and they both know that by bending the pillows things will start to happen. Full of expectation, the girl points at the woman’s pillow, whereby the woman picks up on the invitation and starts to vocalize “TITTITTTITTITIIT” into the corner of the pillow (apparently she knows that this pillow has a microphone). The girl smiles, still full of expectation … The woman says “Now, you play” … But the girl stays still … and awaits. The woman points at the girl’s pillow and shows with hand movements how the girl should bend the pillow to make it respond in sound. The girl then bends the pillow corners while looking at her own hand movements. Then the pillow “says” a slow “TIIIIIITTIIIIITTTITTITIIT”. The pillow’s response comes with two voices, as a mix of a distortion of her close other’s voice and the low voice of a big man. The girl looks at the pillow she keeps in her hands, and the woman looks at the girl, and they both smile. They repeat: the woman says again “TITTITTTITTITIIT” into her pillow, and the girl bends her pillow, without assistance this time, and the pillow responds in the same manner as before. The graphics on the screen is out of their sight and attention, as the two of them repeat taking turns in their playing with the CCTs. All of a sudden the girl’s pillow does not respond in the same way as before; rather it starts to sound like a car with squeaky brakes: SQUEEEEK! This surprises them … and makes them both laugh … It also arouses the girl bodily, and she starts to move her head and upper body rhythmically back and forth, as in a dance*.

The same two children were compared in depth in a study done by Stensæth and Ruud (2012), which I will return to later on in this article, in the “Some empirical observations” section.

## About the RHYME project

In RHYME, which is a 5-year interdisciplinary research project (2010–2015) financed by the Research Council of Norway through the VERDIKT program, the aim is to develop net-based, tangible interactions and multimedia resources that have the potential of promoting health and life quality.^2^ The RHYME research team derives from a collaboration among the fields of interaction design, tangible interaction, industrial design, universal design (UD) and music and health that involves the Department of Design at the Oslo School of Architecture and Design, the Department of Informatics at the University of Oslo, and the Centre for Music and Health at the Norwegian Academy of Music.

In December 2010, the research group submitted a notification form to The Norwegian Social Science Data Services (NSD), which is the Data Protection Official for Research in Norway. In the form, it is discussed how the research group will deal with ethical issues, such as personal and sensitive data. NSD approved the project in February 2011 to gather, secure and store the data according to the standards of ethics in the Norwegian law.

Four empirical studies are to be carried out and three new generations of CCTs are to be developed in collaboration with the Haug School and Resource, the users and the families. So far, the project is halfway along, and two generations have been tested. (More detailed descriptions later on provide examples of the tangibles in use.)

The user-oriented research includes the users’ influence on the development of the media. The users involve six families who have volunteered to participate in RHYME, and the children with disabilities in these six families range in age from 7 to 15 years. All of them become engaged in enjoyable activities when these activities are well facilitated for them. The children vary considerably in terms of behaviour style, from very quiet and anxious to cheerful and rather active. The most extreme outcomes of this variation in behaviour style relate to disability conditions, and mostly those within the autistic spectrum, which applies to four of the children. These conditions include poor (or absent) verbal language and rigidity in their movements. The children’s mental ages range from 6 months to 7 years, and their physical disabilities range from being wheelchair dependent to being very mobile.

One idea behind the RHYME project derives from the development of “An Information Society for All”:

The penetration of ICT in all areas of society enables many groups to gain easier access to public and private services, which paves the way for solutions, which empower many people to live more independent lives and raise quality of life. (2013-06-13: www.regjeringen.no/upload/FAD/Vedlegg/IKT-politikk/stm17_2006-2007_eng.pdf, p. 9)

The ideal here expressed by the state involves a process towards a more inclusive society with equal rights for all citizens (Imrie & Hall, 2001; Iwarsson & Ståhl, 2003; Lid, 2009).^3^ Understood as a democratic issue, such a society would allow for *more* participation for *more* people (Imrie & Hall, 2001; Iwarsson & Ståhl, 2003; Lid, 2009). Interestingly, the last part of the above quotation anticipates the link between independence and quality of life. But is this *necessarily* so, or should we then ask whether, when and for whom independence in fact enhances quality of life? We will return to these questions later on.

An important premise for the following discussion is to understand co-creation as an aspect of participation. The word *participation* derives from the Latin *particeps*, meaning part-taking, and *pars*+*capere*, meaning *to take* or *share in*—that is, in its English iteration, to take part in or become involved in an activity; the state of sharing in common with others; and the act or state of receiving or having a part in something (Simpson & Weiner in Law, 2002). Participation has become a central construct in health care, rehabilitation and various forms of therapy, and it is often used as a way to describe various life areas, such as everyday preoccupations and recreational activities. Participation, in these cases, is often defined as “involvement in a life situation”.^4^

Participation connects to the ideal of UD, which encompasses those broad-spectrum ideas meant to produce buildings, products and environments that are inherently accessible to people both with and without disabilities. Whereas UD research often focuses on the physical standardization of buildings, environments and technology, it seldom addresses user interfaces for alternative and augmented communication *with* tangible and musical health-promoting technology for people with poor verbal language capacity. This is, on the other hand, an agenda of the RHYME project, in the context of the possibilities of co-creation. In the following discussion, co-creation will be presented as an effective means of compromise among several interdisciplinary ideals. Its force as a working concept includes its inherent understanding of how these ideals interrelate.

## Co-creation

*At home we need things to do—together—things that are easily enjoyable and meaningful!* (Mother to a participating child in RHYME)^5^

The simplest way to define co-creation is the act of *creating together*, but this does not address the aspect of health here, either theoretically or practically. What the mother says in the above citation is not only a premise for co-creation but also a basic motivation for this research project: families with a severely disabled child need to have meaningful things to do together at home. Yet this is more complicated than it appears, because what is meaningful to the child with disabilities is not necessarily meaningful to her brother or sister, mother or father.

In these families, it is more than a difference in personal interests that hinders members as they attempt to engage in meaningful activities together. It is also a general difference in approach and in the ability to sustain interest for a prolonged duration. Because the child with disabilities faces the world differently than her family does, it is difficult to establish an ongoing interaction between her and the family members. When a child struggles to concentrate and uses her senses selectively, it is hard for her siblings or parents to share her intentions and maintain their own interest in the interplay with her. The very spark of co-creation is therefore apparent in the above quotation, a mother’s simple but challenging request to come up with meaningful activities that these families can share over time.

## Co-creation interpreted from an interaction design perspective

Cappelen and Andersson, two of the creators behind the CCTs in RHYME, engage with Small’s (1998) notion of *musicking*, with which Small advocates for music as a social activity, a *doing*, rather than a reified object. Andersson (2012) says that the main “doings” in co-creation of music include playing, listening, exploring, composing and collaborating.

*Collaboration* and *play* are particularly crucial to co-creation (Cappelen & Andersson, 2003, 2008, 2011a, 2011b, 2011c, 2011d, 2012), which integrates aesthetic and artistic exploration with play elements (Cappelen and Andersson, 2011a, 2011b, 2011c, 2011d). Eco’s notion of the “open work” as a situation in the process of development also informs Cappelen and Andersson’s thinking:

This [the open work] poses new practical problems by organizing new communicative situations. In short, it installs a new relationship between the contemplation and the utilization of a work of art. (Eco, 1989, p. 56)

Eco’s description of the open work of art as both an active and a passive mode of communication leads directly to Cappelen and Andersson’s development of CCTs as invitations not only to active physical play and cooperation but also to relaxation and meditation as well as recreation and amusement (Andersson, 2012; Cappelen & Andersson, 2003).

### “Human” tangibles?

Cappelen and Andersson describe the CCTs as “smart” and independent *co-creators* (Andersson, 2012; Cappelen & Andersson, 2011a, 2011b). Through the programming of the software, they say, the CCTs can even act like a “human being” (Andersson, 2012; Cappelen & Andersson, 2011a, 2011b). They further describe that because their responses vary, almost like a “musical co-improvizer’s” would, the tangibles behave more like an “independent and flexible play partner”.^6^ This means that when a child plays with the CCTs, he/she does not only get a fixed response each time, as is the case with a traditional musical instrument like a piano or even a synthesizer; rather the CCTs’ responses will vary dynamically over time, with the development in the music as well as with the user interaction based on an earlier interaction. This effect is exemplified in one of the vignettes where the girl and the woman take turns in their playing with the CCTs, and the girl’s pillow suddenly does not respond in the same way as before: From responding with “human-like” voices, the pillow surprisingly responds with a SQUEEEEK, as in the sound of a car braking. The CCTs’ ability to improvize depends on the programming, which can be adapted to individual needs and to the desired degree of “surprise” that is sought.

### Shifting

The characteristic of the CCTs that allows them to resemble improvizing actors is also described as *shifting* by Cappelen and Andersson (2011a, 2011b), here borrowing a notion from Latour (1999). In semiotic theory, which Latour refers to, shifting describes how a person absorbed in reading a text *shifts* to identify with the text’s overall character. Latour specifically calls this *actorially shifting*, as opposed to *spatially shifting* or *temporally shifting*. In the RHYME project, a temporally *direct* (or close) response occurs when the player receives an immediate response to his/her interaction. A spatially or temporally *shifted* response will occur a bit later. The lag time of the temporally shifted response can be programmed and adjusted to suit those children with disabilities who have unusual perceptions of time, for example. Actorial shifting is exemplified in one of the vignettes earlier mentioned: From picking up the woman’s voice in its response, the pillow shifts actorially by taking the “role” of a car.

This quality of shifting not only introduces an element of surprise to the interaction but also involves the player in a fashion that is different from the way musical instruments and toys work. Andersson and Cappelen explain that the CCTs therefore behave more like “improvizing co-musicians or co-players”, and even as “friends and partners in dialogue” (Cappelen & Andersson, 2011a).^7^ That is, the CCTs are not so much what Latour describes as “neutral objects and things” but “mediation” by “immersed interactive players” (Latour, 1999). The player thereby projects meaning and actions into the CCTs by empowering them, according to Cappelen and Andersson, as “actors” with the potential to influence the player in turn:

The shifted response invites the user to shift position spatially, temporally and role based, or actorially, during the interaction. The possibilities to shift at all times make it possible for the user to dynamically choose the activity level, and role to play, no matter if he wants to be the person driving the action further on, or to take a more relaxed spectator role in an ambient physical environment. (Cappelen & Andersson, 2011a, pp. 189–193)

A crucial aspect of co-creation is therefore the fact that the roles of the actors (people, objects) can change. Instead of remaining self-focused on his/her own playing with and controlling/mastering of a given instrument or toy, the child with disabilities and/or his/her siblings can relate to the CCTs as active and exciting play partners.

### The programming code and the musical processes

To explain co-creation further we must also look at the *programming code*, which is written by the composer:

Instead of writing one linear work, he creates an infinite amount of *potential music* that reveals itself through various answers to user interactions in many situations based on *genre* and music knowledge and competence in social behavior (Cappelen & Andersson, 2008, p. 84, emphasis in the original)

Three levels occur in this potential music, as Cappelen and Andersson describe it: (1) sound nodes, (2) algorithm and (3) narrative structure. The sound nodes are the least-defined musical entities—tones, chords and rhythmic patterns. They can occur as sequences or parallel events throughout the algorithm. The user is able to experience the output response as phrases and narrative structures based on one of the programmed genres (Cappelen & Andersson, 2008). Algorithm includes the programming of the *composition rules* and the *interaction rules*. The composition rules combine the sound nodes, and the interaction rules are the computers’ treatment of the users’ interactions. The interesting aspect is that the computers do not treat the interactions mechanically, as a piano for example would do. Rather they treat the interactions dynamically; they are based on the user interactions over time and the composition rules, which in turn are based on aesthetics and/or musical genres and the narrative structure over time. It is this use of the computers’ dynamic capacities that makes it possible for the CCTs to vary and shift their responses.

The radical aspect here is that the child with disabilities, via his/her intervention using the interactive tangibles, can manipulate the rules of composition and the musical processes. The CCTs will then respond to his/her intervention by creating a new musical narrative. At the same time, the visual design on the screen/wall changes too. Over time, this narrative structure will create expectations of potential future musical outputs with variations, so that the user can negotiate the shaping of the musical narrative and promote its musical interest at the same time. This is what makes the musical tangibles in RHYME more *co-creative* than other responsive interactive musical devices, such as Sound Beam and Midicreator (see, for example, Magee & Burland, 2008, www.soundbeam.co.uk) or the lesser-known Jam2jam tool (Adkins, Summerville, Knox, Brown, & Dillon, 2012). Therefore, when compared to traditional musical instruments, such as piano and drums, the CCTs in RHYME feature expanded roles (Cappelen & Andersson, 2011b).

In summary, an outline of co-creation from an interaction design perspective highlights the *collaboration* between the CCTs and the child with disabilities. As we shall see below, other perspectives have different priorities.

## Co-creation in music and health perspectives

The prefix in *co*-creation emphasizes not only *co*llaboration but also *co*mmunication, which comes from Latin *communicare*, meaning, “to share, divide out; impart, inform; join, unite, participate in”, lit. “to make common” (from communis) (www.dictionary.reference.com/browse/communication?s=t). From the perspectives of music and health, as represented by the present author, a music therapist, and her colleague Even Ruud, both researchers in the RHYME group, the greatest potential of CCTs is as a means of communication. In another study (Stensæth & Ruud, 2012), we assert that co-creation encompasses *communicative sharing*, which converts CCTs into social tools dedicated to well-being and life quality. This is especially relevant given the degree to which the CCTs afford interpersonal interaction and the sharing of meaningful experiences between subjects, and between the subjects and the objects. From this perspective, then, the focus is mainly on the relationship between the child with disabilities and a “close other”.^8^ This change of focus draws attention to other aspects of co-creation, as we will see below.

### Relation

From a music and health perspective, the *relation* is in itself seen to have health potential (Stensæth, 2008; Trondalen, 2008). Trondalen (2008) links relation to what she calls “intersubjective moments of meeting”.^9^ This kind of intersubjectivity, as she describes it, is a relational dimension between two people that hosts aspects such as emotional sharing, mutual recognition and common focus. During the process of mutual recognition, in particular, there can emerge a symbolic space that enables attunement and mirroring, and Trondalen labels this space “thirdness”.

Benjamin, an influence upon Trondalen, similarly anticipates aspects of this discussion in his prescient term “co-created third”:

The thirdness of attuned play resembles musical improvisation, in which both partners follow a structure or pattern that both of them simultaneously create and surrender to, a structure enhanced by our capacity to receive and transmit at the same time in nonverbal interaction. The *co-created third* has the transitional quality of being both invented and discovered. To the question of “Who created this pattern, you or I?” the paradoxical answer is “Both and neither”. (Benjamin, 2004, p. 7, my emphasis)

Thirdness directly addresses the possibility that the relationship holds more than what two people bring to their interaction—it invites them to be simultaneously aware of “me”, “you” and “us” (Benjamin, 2004). A music and health perspective upon RHYME encompasses all of the mechanisms involved in the collaboration between the CCTs and the users that might enhance health.^10^

### Health musicking

By incorporating the perspective of musicking and relation (see earlier paragraphs) the definition of co-creation as a notion is broadened to include actions and doings as well as intersubjective moments of meetings. This means that the users and the CCTs actively create *meaningful experiences* together (Stensæth, 2008; Trondalen, 2008).

In order for musicking to become health promoting, we need to add health to it, and *health musicking* (Stige, 2012), which is not new, is a notion that seems meaningful to apply to our definition of co-creation. Health is a tricky concept, of course. WHO (2007/2001/1948) defines health as a state of complete physical, mental and social well-being and not merely the absence of disease or infirmity. In addition to the further thoughts about health in this text, positive psychology also broadens the notion of health by drawing our attention to the nurturing of the positive aspects of life in tandem with the treating of disabilities or illnesses (Seligman & Csikszentmihalyi, 2000).

Health musicking also includes a *salutogenic* orientation, which focuses on factors that support human health and well-being, rather than on factors that cause disease. Antonovsky’s (1987) notions of health as a personal experience (and an ongoing process), rather than a biomedical state, inspire this orientation. Antonovsky (1987) connects health to the extent to which we perceive the world as *making sense*, and to our interest in experiencing a sense of coherence there.

Additionally, health musicking evokes Aldridge’s (2004) description of health as *performance*. Aldridge claims that “becoming healthy” is an intentional act aimed at balancing physical, psychological and social elements to create or enhance well-being and quality of life. This way, health musicking becomes a “provider of vitality” (Bonde, 2011; Ruud, 2010), or “a tool for developing agency and empowerment; a resource or social capital in building social networks; a way of providing meaning and coherence in life” (Ruud, 2010, p. 111). In the RHYME project, the performativity of co-creation is extremely salient, whether via companionship, actual co-performance. Thus, health musicking relates to co-creation as both a social and an individual practice through which people use music experiences to create meaning and coherence in times of adversity (see also Bonde, 2011). We could therefore say that health musicking comes to encompass all of the ways in which music experiences provide health affordances.

The measurement of these potential health benefits, of course, requires us to observe the CCTs in use, which is the reason for the RHYME project. Do the CCTs counteract isolation by affording health musicking? More concretely, does the data reveal health-promoting activities and interaction among children with disabilities and their close others? Before we return to a more theoretical discussion of co-creation, we will next review some empirical observations related to CCT’s affordances from Stensæth and Ruud’s (2012) study.

## Some empirical observations

Based on video analysis of two of the six participants with disabilities, namely the boy and the girl already presented in the vignettes, Stensæth and Ruud looked at how these children related to the CCTs in a potentially health-promoting fashion. The observations derived from the first rounds of data collection, which include four sessions of 30 min each, was done with the interactive installation called ORFI (see www.RHYME.no). We need to say that ORFI had been made before the RHYME project started, and that the research group related to these CCTs as a basis for discussion and development of the new CCTs.^11^

**Figure:**
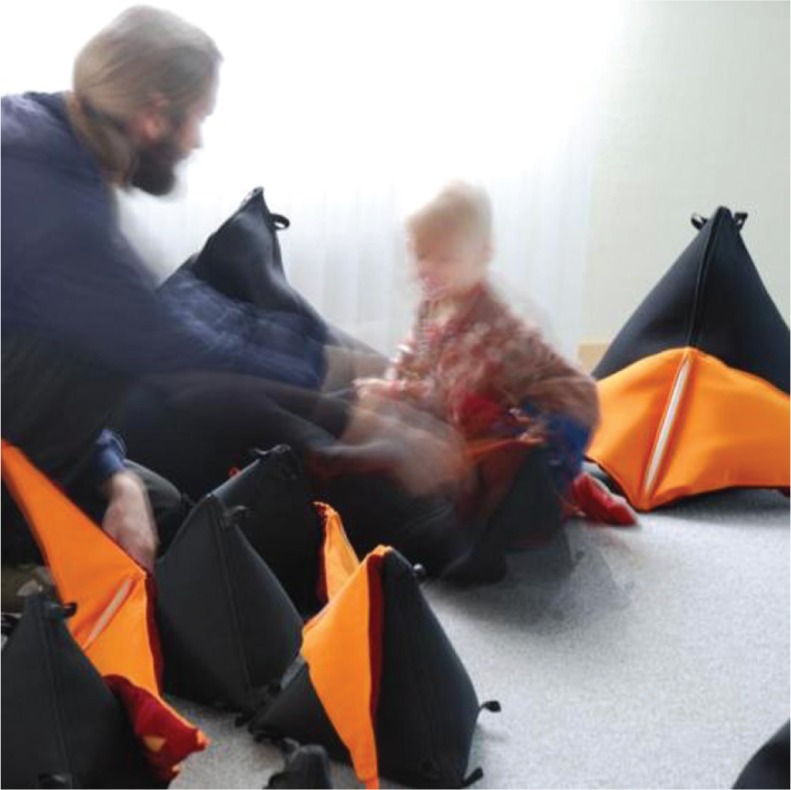


ORFI consists of 26 soft pyramid-shaped modules of “pillows” in three sizes ranging from 30 to 90 cm on a side. The ORFI installation contains eight different musical and graphical genres (see www.rhyme.no for information on the genres). Two modules contain microphones that produce live music based on their recordings of the participants’ own voices and environmental sounds. Most of the modules are covered in black cloth with orange “wings” with sensors inside that react when being bended - and lights along one side. Some modules contain audio speakers. When a user touches, sits on or cradles one of these modules, they will feel its vibrations. Every module contains a microcomputer and a radio device, so they are able to communicate wirelessly with one another. To interact with the modules in ORFI, the user must bend the wings, which generate prompt alterations in the music, and the lights on the pillows (Cappelen & Andersson, 2011d).

**Figure:**
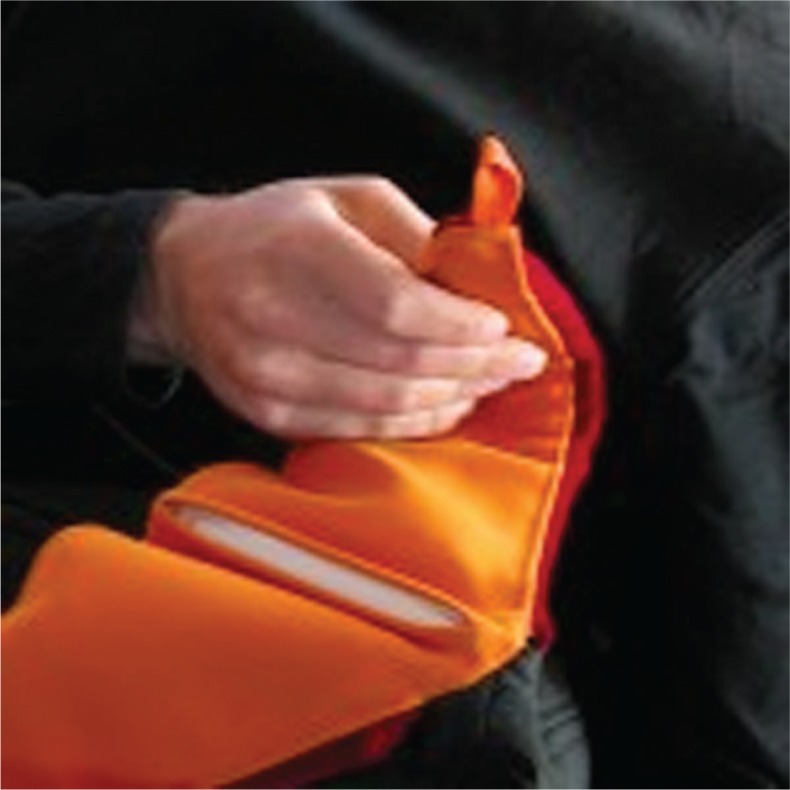


ORFI also generates graphics that can be projected on the wall or on a screen:

**Figure:**
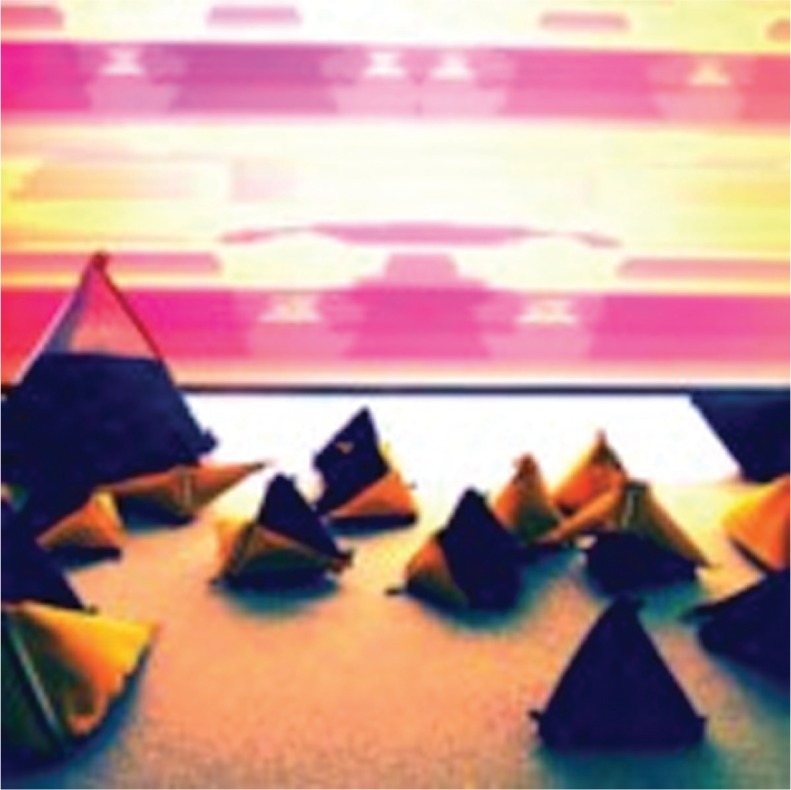


### Observations from a case study

In the interests of exploring various uses of the CCTs in the ORFI installation, the boy and the girl with rather different behavioural styles and approaches, were compared in depth. As we have already learned from the vignettes, the boy was rather active physically, laughing and throwing his body around while playing with ORFI. The girl, who needs a wheelchair, was physically quieter, but showed great interest in exploring the modules while either sitting or lying on the floor, sometimes rocking to the music while touching and bending the wings. She also used her voice while exploring ORFI.

The children came to the activity with close others from the school they attend. Each child approached the CCTs in a personal way that evoked how each child faced the world as well. The boy, for example, wanted to be “in charge” and initially focused his attention on his close other to ensure her interest in and response to his initiatives. Then he became more absorbed by the tangibles, which in turn lessened his need to control his close other. The girl was initially more open to possibilities—when the tangibles and activities were inviting enough, she was soon absorbed in the playing and exploring.

### Vitalization

The health potentials revealed by the analysis showed that the CCTs were able to *vitalize the children bodily and mentally*:

1. The children seemed to be *stimulated to explore through their basic senses*, such as hearing, sight, tactile sense, kinesthetic sense, proprioceptive sense and vestibular sense;
2. their *mastery and sense of agency seemed to be strengthened*, which
3. afforded the children new “possibilities of actions” (see Ruud, 1998) *and* “new possibilities of interaction” (see Stensæth, 2008), which in turn
4. *empowered them* to become creatively and aesthetically engaged.

Vitalization, an activation of the various levels and intensities of one’s vitality, was particularly present here and carried the greatest health potential. The fact that the children displayed their own personal styles in their engagements with the CCTs is also significant. Both of these aspects correspond to an ecological perspective on health, whereby health is an ongoing (active) process that needs to be continuously renewed. As Bruscia (1998) observes, ecological health involves the actualization of “one’s fullest potential for individual and ecological wholeness” (p. 84).

Along with vitality, we observed communication in the two children through

- contact with themselves and the feeling of a subjective self;
- contact with the CCTs and with their co-creative close others (intersubjective aspects),
- developing relations to subjects, objects and environment, and
- *qualitative sharing* of feelings (interaffectivity) (see Stensæth & Ruud, 2012).

The above aspects, in particular those connected to intersubjectivity and interaffectivity, clearly relate to Daniel Stern’s elaboration on *dynamic forms of vitality* (Stern, 2010), which not only refers to the inner experiences of being alive; they are also always present for interpersonal relation. Therefore, they are connected to one aspect of communicating and understanding any temporally based human activity evoking a felt experience in another being (Stern, 2010). This reminds of what Trondalen (2008) earlier on referred to as “intersubjective moments of meeting”.^12^

In addition, the analyses showed that the CCTs’ shifting, which is the quality that differentiates them from many other interactive toys and instruments, created much joyful expectation in the children. Along with the tactile qualities, music and graphics, this shifting attracted the children greatly. One could say that the children seemed to presuppose the CCTs to behave in a “human” way (as initially intended and expressed by their creators) in that they expected the tangibles to improvize and surprise.

## Critique of the empirical observations

In the first rounds of participant actions, we hoped to prepare a detailed list of what occasioned a positive response to the CCTs. Because we were exploring the tangibles’ *potentials*, we realized many positive results, but there were problems with the data collection in RHYME as well.

Sometimes, for example, it was difficult for the children to know what to do or how to do it with the CCTs. The idea of the CCTs as an “open work” in the sense of Eco is good in theory but less so in practice, where something can be *too open* if it affords little in the way of instructions or prompts for possible interactions. Another challenging aspect was that when the computers failed, which happens sometimes with vulnerable equipment, the children and their close others were not able to restore the media on their own. This happened only once or twice during the described participant actions, but the creators of the CCTs, who were there, soon fixed the problem. Without them a computer failure would have been a big challenge. Together with the fact that the CCTs seemed rather advanced in character, we anticipated that the distraction of technical details could lead to loss of interest in the CCTs and even a larger sense of resignation among the children, which is not the intention of the RHYME project. However, we must remember that ORFI was initially not developed for children with disabilities and is primarily used in the RHYME project as a basis for discussion and development.^13^

The most obvious criticism of this first round of actions was that most of the observations sessions were conducted in a school setting, which likely meant that the participants anticipated an educational outcome from playing with the CCTs. In addition, the close others were most often skilled professionals, and we wondered whether they were sometimes too active or clever compared to an everyday CCT’s situation. In a home setting (for which the CCTs are ultimately intended and which we have yet to test), the CCTs are more likely to be perceived simply as “playful furniture” for recreational use. Also, at home, with siblings, for example, we would expect that the co-creation might be either quieter or wilder.

A further crucial point, or problem, was that the children seemed not to be very *independent* in their activities with the CCTs. Instead, they explored the tangibles very much *through* their close others and required a lot of physical and/or psychological support as they interacted. In other words, the most effective access to the CCTs was through other human beings. But was this in fact a bad thing?

## Discussion

To answer the question, we must look past the objects to subjectivity: Instead of asking what it is that makes physical objects accessible for human beings, which would be inherent to a UD perspective, we need to ask what it is that makes *human beings accessible*, to themselves and to one another? It seems meaningful to address these questions by first relating co-creation to philosophical aspects of dialogue.

### Co-creation as dialogue

“Der Mensch wird am Du zum Ich”, said Buber in 1923/1965. In this famous quote Buber points that we move into existence in the “I” towards “Thou”, which is a relationship without bounds. The I–Thou relation is a concrete encounter, because the two beings meet one another in their authentic existence, without any qualification or objectification of one another (Buber, 1923/1965). Bakhtin (1981), who was inspired by Buber, called the I–Thou relationship a basis for existence. He said:

“To be” means to be for another, and through the other, for oneself. A person has no internal sovereign territory, he is wholly and always on the boundary; looking inside himself, he looks into the eyes of another with the eyes of another. (Bakhtin, 1984, p. 287, my quotes)

In a fundamental way, dialogue intersects with life itself; it does not exist without people and their interaction. Existence then becomes the event of co-being, which manifests itself in the form of a constant, ceaseless creation and exchange of meaning. “Being” for Bakhtin is therefore simultaneity of a co-being. Transferred to RHYME we could say that the users not merely co-create as a way to express themselves but also to communicate and to be *in* dialogue (Bakhtin, 1986; Stensæth, 2008). Co-creation then becomes a way to ensure a feeling of co-being (see also Stensæth, 2008, 2010).

We have seen that for some children with disabilities, like the two described in this article, co-creation sometimes requires other people, either for indirect support—for example, as a person who shares and recognizes their experiences—or for direct support, as the pivotal “missing link” between the children and the objects. In the latter case, we might say that the close other becomes herself a technology for co-creation, as a premise for the child’s very ability to respond. We might then say that the close other makes the child with a disability *response-able*, in the most literal sense.^14^ The role of the close other is therefore sometimes vital for ensuring co-creation in order for the child with disabilities to share-in and participate.

Matell (2011), who discusses the concept of participation in relation to music therapy, asserts that human beings require *social participation*. If this participation is denied, human beings tend to develop strategies (such as aggressive or destructive behaviour) to compensate for their isolation. For the boy and the girl presented in this article, isolation is a real possibility, and co-creation could be seen as a label that *prevents* both destructive and isolating preconditions. For them isolation is something that has to be overcome and dialogue is something that needs to be established in order for co-creation to happen.

Dialogue, as we have described it so far, also resonates well with research on early infant interaction, another model for understanding co-creation in relation to a social participation. It has been shown that we are all born to be sociable—to both communicate and share meaning (Stensæth & Trondalen, 2012; Trevarthen & Malloch, 2000). In the context of theories from Buber, Bakhtin, Malloch and Trevarthen, I would propose that, on the one hand, there is a human capacity to communicate and share experiences (that is, to co-create), regardless of the presence of a disability, but on the other hand, we must be allowed at least the possibility of using this capacity. The human being is born to *seek* intersubjectivity and make cultural learning in companionship possible (Matell, 2011; Stensæth & Trondalen, 2012). In the same way, we could say that the users in RHYME *seek* to co-create through companionship with the close other, and that the CCTs offer a space—or a field—in which the co-creation can take place.

To sum up this discussion, we could say that Stensæth and Ruud’s (2012) study is useful in that it generates insight into how micro-level experiences of intersubjectivity relate to broader theories. Co-creation could therefore be viewed as a social model that encompasses environmental factors ranging from the individual’s most immediate environment to the general environment (including both social and institutional structures). This model further draws attention to the ethical commitment that we all have to face other people dialogically, whether they have disabilities or not. Action is the primer. We could say with Bakhtin (1986) that co-creation requires action, not in the sense of problem solving, but in the sense of relating. Eventually, in co-creation, action insists on a *co-action in joint attention* and being actively engaged, face-to-face, in a live situation.

### A synthesis of perspectives

We have seen that an interaction design perspective emphasizes the collaboration between the child with disabilities and the CCTs, whereas the music and health perspective emphasizes the collaboration between the child with disabilities and the close other. We have also learned that for co-creation to afford health musicking, we must allow for *combinations* of collaborations among all of the CCTs, the child and the close other. For some children, such as the boy and girl above, it is the relation between the child and the close other that creates the most effective collaboration with the CCTs. In the future, when the CCTs are tested at home within core families, it may be that a brother’s particular interaction with the CCTs that can promote collaboration between him and a sibling with disabilities.

**Figure 1:**
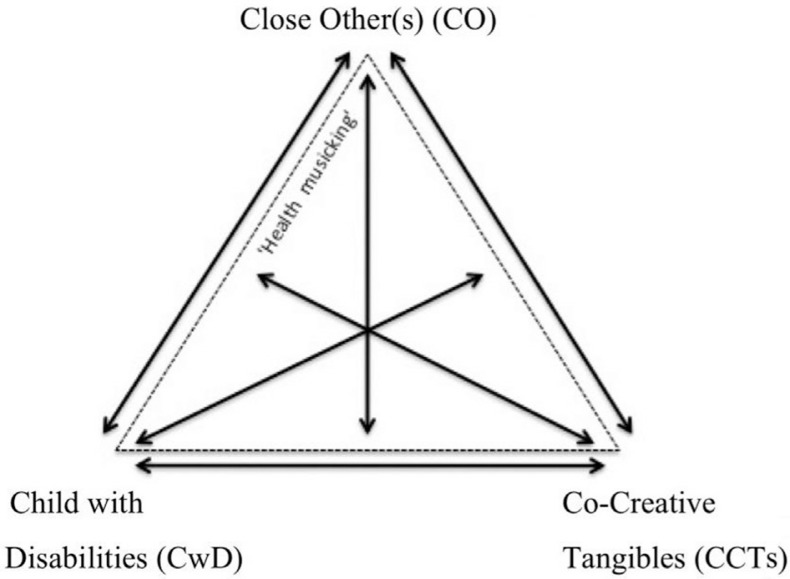
Combinations of collaborations that can come in to play between the child with disabilities, the close other, and the co-creative tangibles. The arrows point at combinations of collaborations.

Figure 1 suggests a model that represents many potential combinations of collaboration in co-creation:

The triangle has three corners. The three actors, CO, CwD and CCTs, are each placed at a corner. The arrows outside the triangle show possible collaborations between the actors in each corner; they can also be understood as *relations* and consequently as units that can in turn collaborate with another actor in another corner. The arrows inside the triangle show what these potential collaboration combinations are:

- The relation between the CwD and the CCTs collaborates with the CO.
- The relation between the CwD and the CO collaborates with the CCTs.
- The relation between the CO with the CCTs collaborates with the CwD.

Health musicking comes about both inside and outside the triangle, hence the dotted lines. However, as we have discussed, for co-creation to be effective, it is often the area *inside* the triangle that is activated.

There are also more complex collaboration combinations. Sometimes, when the child with disabilities depends on the (direct or indirect) support of a close other, it is their relation that collaborates with the CCTs. In this case, as mentioned above, the close other is almost himself/herself a technological prerequisite for the child’s co-creation.

Moreover, the various collaboration combinations are both *flexible* and *situated*. This means that if we want to measure life quality, we must always ask if, when and for whom and make sure that the individual child’s needs comes before the UD ideal of independence. The same people can create various collaboration combinations in different situations, and the intensity and level of co-creation will vary. For example, when the child with disabilities has a tough day (physically or/and mentally), he/she can be more dependent upon close others. It is also true that sometimes it is simply more fun to explore the relation than the CCTs (or the other way around). Then, one collaboration combination will supersede the others. Often, as well, several collaboration combinations will be in play.

Over time, it is likely that experienced and embodied collaboration combinations pave the way for other collaboration combinations. The child with disabilities, having co-created intensively with his/her brother, might then expect more intense co-creation with his/her close other as well. These renewed collaboration combinations appear in the minds of the co-players as memories and images, or even as containers of “something third” that were explored in the past.

## Conclusion

The research question, again, was as follows: What does co-creation imply when musical and interactive tangibles are used as health-promoting implements for families of children with disabilities?

First, co-creation implies health musicking, which incorporates the family’s desire *to do* (action) *something* (activities) *meaningful* (intentional) together (*intersubjective and interpersonal*). This is an ecological aim in that it implies the process of continuously promoting health while also preventing poor health. It also implies a strengthening of agency and mastery, as well as the creation of embodied, sensory and empowering interactions with both the tangibles and other people.

Co-creation further implies intersubjective communication, which requires a dialogical mindset in the users. This mindset comes natural and is not something the child with disabilities or her family is aware of while they are collaborating via the CCTs. However, knowledge from music and health—and music therapy, including especially those perspectives that are influenced by participation philosophy and early interaction psychology—is useful for the design of future CCTs. This knowledge informs how human beings make themselves accessible towards each other and towards objects.

We have seen that, in the context of RHYME, it is valuable to design and program the CCTs so that they behave in a more human fashion. Empirical observation also reveals that their shifting makes the CCTs fun, desirable, erratic and surprising in their responses. In this way, the objects too respond somewhat dialogically, so that users can share actions and feelings *through* and *imaging with* the CCTs.

Finally, as an image of health-promoting participation, we might wonder whether this outlining of co-creation approaches what Stige (2006) calls a “right to play” (p. 115) via activities distinguished by “cohesion, solidarity” and “high levels of emotional support” (loc. it) from potentially close others? Co-creation might then afford an alternative perspective on participation and democratization as well.

## Conflict of interest and funding

The author has not received any funding or benefits from industry or elsewhere to conduct this study.

## Footnotes

1 The idea in this article is not to describe the children and their diagnosis in detail; rather it is to study how *co-creation* is stimulated, either by evoking feelings in the children, or accommodating their needs to act and to create social relations. For more detailed information about the data collection, see www.RHYME.no.

2 The design of these interactions and resources is presented later on. See also www.rhyme.no.

3 In Norway, there is already a Minister of Inclusion.

4 “Involvement in a life situation” is by the WHO (2001) categorized into domains like learning and applying knowledge; general tasks and demands; and communication, social and civic life. WHO proclaims that, in the International Classification of Functioning, Disability and Health (ICF), participation has moved away from being a “consequences of disease” classification (WHO, 1980 version) to become a “component of health” classification (WHO, 2001, p. 4).

5 The mother said this in an interview done by the research group with all of the participating families in 2011. For more about the data collection methods, see www.RHYME.no.

6 These are Andersson’s and Cappelen’s words and descriptions.

7 Again, these are Andersson’s and Cappelen’s words.

8 Because the child with disabilities is generally accompanied by a family member or assistant (such as from the special education school where some of the research actions were carried out), the co-player will be called a “close other”.

9 Trondalen (2008) also calls these “significant moments”—significant to the therapy, that is.

10 Some of these mechanisms can have *therapeutic potential*, however. How RHYME relates to music therapy is an issue for another time.

11 ORFI was first made by the Birgitta Cappelen, Anders-Petter Andersson, and Frederik Olofsson in Musicalfieldsforever (see www.musicalfieldsforever.com), which is the name of both a group and a project.

12 Sterns’s theories have inspired Tondalen greatly.

13 Some of the challenges and problems describe above have been dealt with in the development of the next generation of CCTs; the WAVE installation (see: rhyme.no/?page_id=1034).

14 In my dissertation (Stensæth 2008), I discuss “musical answerability” in the context of defining music therapy improvization (which encompasses all of the relations among therapist, client and music). Music therapy improvization is a means through which to transform isolated human utterances into intentional communicative expressions. I borrow “answerability” from Bakhtin, who uses it early on, before he settles upon the term “dialogue”.

## References

[CIT0001] Adkins B, Summerville J, Knox M, Brown A. R, Dillon S (2012). Digital technologies and musical participation for people with intellectual disabilities.

[CIT0002] Aldridge D (2004). Health, the individual and integrated medicine.

[CIT0003] Andersson A.-P (2012). Interaktiv musikkomposition.

[CIT0004] Antonovsky A (1987). Unravelling the mystery of health–How people manage stress and stay well.

[CIT0005] Bakhtin M (1981). The dialogic imagination.

[CIT0006] Bakhtin M (1984). Problems of Dostoevsky’s poetics.

[CIT0007] Bakhtin M (1986). Speech genres and other late essays.

[CIT0008] Benjamin J (2004). Beyond doer and done to: An intersubjective view of thirdness. Psychoanalytic Quarterly.

[CIT0009] Bonde L. O (2011). Health music(k)ing—Music therapy or music and health? A model, eight empirical examples and some personal reflections. Music and Arts in Action (special issue: Health promotion and wellness).

[CIT0010] Bruscia K (1998). Defining music therapy.

[CIT0011] Buber M, Buber M (1923/1965). Ich und Du [I and Thou]. Das dialogische Prinzip [The dialogical Principle].

[CIT0012] Cappelen B, Andersson A.-P (2003). From designing objects to designing fields—from control to freedom. Digital Creativity.

[CIT0013] Cappelen B, Andersson A.-P (2008).

[CIT0014] Cappelen B, Andersson A.-P (2011a). Design for co-creation with interactive montage.

[CIT0015] Cappelen B, Andersson A.-P (2011b). Expanding the role of the instrument.

[CIT0016] Cappelen B, Andersson A.-P (2011c). Designing smart textile for music and health. Ambience 11.

[CIT0017] Cappelen B, Andersson A.-P (2011d). The empowering potential of re-staging. LEA (Leonardo Electronic Almanac).

[CIT0018] Cappelen B, Andersson A.-P (2012). Musicking tangibles for empowerment. ICCHP2012 Proceedings.

[CIT0019] Eco U (1989). The open work.

[CIT0020] Imrie R, Hall P (2001). Inclusive design: Designing and developing accessible environments.

[CIT0021] Iwarsson S, Ståhl A (2003). Accessibility, usability and universal design: Positioning and definition of concepts describing person-environment relationships. Disability and Rehabilitation.

[CIT0022] Latour B (1999). Pandora’s hope: Essays on the reality of science studies.

[CIT0023] Law M (2002). Distinguished scholar lecture: Participation in the occupations of everyday life. American Journal of Occupational Therapy.

[CIT0024] Lid I. M (2009). Hva kan man oppnå gjennom universell utforming? En undersøkelse av ulike sider ved begrepet. FORMakademisk.

[CIT0025] Magee W, Burland K (2008). An exploratory study of the use of electronic music technologies in clinical music therapy. Nordic Journal of Music Therapy.

[CIT0026] Matell M (2011). Mutual participation in and through music therapy for children with visual impairment.

[CIT0027] Ruud E (1998). Improvisation, communication, and culture.

[CIT0028] Ruud E (2010). Music therapy: A perspective from the humanities.

[CIT0029] Seligman M. E. P, Csikszentmihalyi M (2000). Positive psychology. An introduction. American Psychologist.

[CIT0030] Small C (1998). Musicking: The meanings of performing and listening.

[CIT0031] Stensæth K (2008). Musical answerability: A theory on the relationship between music therapy improvisation and the phenomenon of action.

[CIT0032] Stensæth K, Stensæth K, Eggen A. T, Frisk R (2010). Å spele med hjartet i halsen. Musikk, helse, multifunksjonshemming.

[CIT0033] Stensæth K, Ruud E (2012). Interaktiv helseteknologi—nye muligheter for musikkterapien?. Musikkterapi.

[CIT0034] Stensæth K, Trondalen G, Trondalen G, Stensæth K (2012). Interview with Stein Bråten and Colwyn Trevarthen. Barn, musikk, helse.

[CIT0035] Stern D (2010). Forms of vitality: Exploring dynamic experience in psychology, the arts, psychotherapy, and development.

[CIT0036] Stige B (2006). On a notion of participation in music therapy. Nordic Journal of Music Therapy.

[CIT0037] Stige B, MacDonald R, Kreutz G, Mitchell L (2012). Health musicking: A perspective on music and health as action and performance. Music, health, and wellbeing.

[CIT0038] Trevarthen C, Malloch S. N (2000). The dance of wellbeing: Defining the musical therapeutic effect. Nordic Journal of Music Therapy.

[CIT0039] Trondalen G, Trondalen G, Ruud E (2008). Den realsjonelle vending—Fra en-person til to-person [The relational turn—from one-person to two-person]. Perspektiver på musikk og helse. 30 år med norsk musikkterapi [Perspectives on music and health. 30 years with Norwegian music therapy].

[CIT0040] World Health Organisation (WHO) International classification of functioning, disability and health—children and youth (ICF-CY).

